# Editorial: Lifestyle and vascular ageing

**DOI:** 10.3389/fspor.2023.1249268

**Published:** 2023-07-13

**Authors:** Karsten Königstein, Christopher Klenk, Jannik Sonnenberg, Lukas Streese

**Affiliations:** ^1^ Division Sports and Exercise Medicine, Department of Sport, Exercise and Health, University of Basel, Basel, Switzerland; ^2^ Department of Diagnostic and Interventional Radiology, Klinikum Rechts der Isar, Technical University of Munich, Munich, Germany; ^3^ Department of Medicine B, Gastroenterology and Hepatology, University Hospital Münster, Münster, Germany; ^4^ Faculty of Health Care, Niederrhein University of Applied Sciences, Krefeld, Germany

**Keywords:** early vascular ageing, lifestyle, cardiovascular health, blood pressure, diet, physical activity

**Editorial on the Research Topic** Lifestyle and vascular ageing

Vascular ageing is a lifelong process, and cardiovascular diseases are the result of genetic predisposition, fetal programming, and environmental factors (1). Individual trajectories vary from early vascular ageing (EVA) to supernormal vascular ageing (SUPERNOVA) (2). While EVA is characterized by premature vascular dysfunction and structural remodeling and associated with an increased burden of cardiovascular diseases, SUPERNOVA is the exact opposite. It is defined as extremely low stiffening and high elasticity of the arteries until old age associated with extremely low cardiovascular morbidity.

The prevention of EVA needs to comprise subjects along the entire life span starting with pediatric populations. A study of 642 Mexican children and adolescents aged from 6 to 19 years observed an 8.0% prevalence of prediabetes (Lares-Villasenor et al.). More time playing video games, a surrogate of sedentary behavior, was not associated with higher blood glucose levels. However, higher socioeconomic status and higher healthy eating index were associated with lower blood glucose levels. These observations demonstrate an association between lifestyle behavior and cardiovascular risk exposure already in young children. Further evidence suggests that the improvement of cardiovascular risk factors in pediatric populations via lifestyle modifications may lead to a more favorable vascular phenotype in adulthood, thus reducing the likelihood of cardiovascular events (3).

It is beyond doubt that high cardiorespiratory fitness and regular physical activity exert beneficial effects on cardiovascular risk factors (4) and also directly on the vascular organ (5). Vascular biomarkers of endothelial function, arterial stiffness, and wall thickness are used to predict the effects of lifestyle and cardiovascular risk factors on vascular function and structure. The authors of a large population-based study on Chinese adults demonstrated the impact of mean arterial blood pressure on age-related changes of the brachial–ankle pulse wave velocity within a 5-year observation period (Deng et al.). This study highlights the pressing need for more projects creating trajectories of vascular biomarkers to understand the impact of lifestyle-based risk factor modification on vascular ageing.

However, the associations of cardiorespiratory fitness and physical activity with biomarkers of vascular function and structure are often heterogeneous. For example, a study on a sample of older Brazilian adults found variable associations of moderate-to-vigorous physical activity and performance in a 6 min walking test, a surrogate of cardiorespiratory fitness, with aortic pulse wave velocity, a marker of arterial stiffness, and with carotid intima-media thickness (De Sousa et al.). The authors showed that higher levels of cardiorespiratory fitness but not of moderate-to-vigorous physical activity were associated with better vascular health phenotypes in 82 older individuals (mean age 67 ± 5 years). This study highlights the protective effect of high cardiorespiratory fitness levels on vascular health and contributes to the important message that cardiorespiratory fitness is a strong predictor of end-organ damage and mortality (6). Such observations add to a mounting body of evidence suggesting that the characterization of vascular health and its modifiability is likely to be a matter of an integrated understanding of multiple clinical and molecular biomarkers rather than the result of the assessment of a single indicator.

Over the past 30 years, the important role of lifestyle behaviors for the individual course of vascular ageing has become very clear. However, the worldwide incidence of physical inactivity, sedentary behavior, and unhealthy diet keeps increasing (7, 8). This calls for effective countermeasures to support lifelong health in our ageing population (9). Efforts to promote population-wide healthy vascular ageing need measures of public education that raise the awareness of the harms and benefits associated with changes in lifestyle behavior. Politically guided national policies and incentives for relevant companies pave the way for the development and application of effective measures of cardiovascular prevention. In this context, an up-to-date and sophisticated review has offered an integrative approach to bringing clinical knowledge into public strategies to reduce salt intake among the Chinese population (Jiang et al.). The authors emphasize the need to customize such strategies based on cultural peculiarities in the respective country.

Despite such population-wide measures, primary and secondary vascular prevention require personalized approaches to individual medical care. (Kubiak et al.) evaluated the role of the advanced nurse practitioner in the care of patients with peripheral artery disease in France. By consulting the patient after hospital discharge at home, the advanced nurse practitioner ensures clinical assessment, nursing supervision, adverse event screening, and renewing of drug prescriptions. This concept extends in-hospital patient care into the home-based environment and is in line with the current policy of the P4 medical approach to individual care (10).

In summary, the collection of articles published under this research topic addresses the need for more scientific evidence to improve (a) the understanding of the mechanisms underlying the effects of lifestyle on individual vascular ageing, (b) the identification and application of novel and established biomarkers that sensitively monitor vascular lifetime trajectories with and without treatment, and (c) both population-wide and individual medical programs for an effective implementation of healthy lifestyle behaviors.
